# Bariatric surgery improves postprandial VLDL kinetics and restores insulin-mediated regulation of hepatic VLDL production

**DOI:** 10.1172/jci.insight.166905

**Published:** 2023-08-22

**Authors:** Vehpi Yildirim, Kasper W. ter Horst, Pim W. Gilijamse, Dewi van Harskamp, Henk Schierbeek, Hans Jansen, Alinda W.M. Schimmel, Max Nieuwdorp, Albert K. Groen, Mireille J. Serlie, Natal A.W. van Riel, Geesje M. Dallinga-Thie

**Affiliations:** 1 Department of Public and Occupational Health, Amsterdam University Medical Centers, Amsterdam, The Netherlands.; 2 Department of Mathematics, Erzurum Technical University, Erzurum, Turkey.; 3 Department of Endocrinology and Metabolism, and; 4 Department of Experimental and Vascular Medicine, Amsterdam University Medical Centers, Amsterdam, The Netherlands.; 5 Department of Biomedical Engineering, Eindhoven University of Technology, Eindhoven, The Netherlands.

**Keywords:** Metabolism, Insulin, Lipoproteins

## Abstract

Dyslipidemia in obesity results from excessive production and impaired clearance of triglyceride-rich (TG-rich) lipoproteins, which are particularly pronounced in the postprandial state. Here, we investigated the impact of Roux-en-Y gastric bypass (RYGB) surgery on postprandial VLDL_1_ and VLDL_2_ apoB and TG kinetics and their relationship with insulin-responsiveness indices. Morbidly obese patients without diabetes who were scheduled for RYGB surgery (*n* = 24) underwent a lipoprotein kinetics study during a mixed-meal test and a hyperinsulinemic-euglycemic clamp study before the surgery and 1 year later. A physiologically based computational model was developed to investigate the impact of RYGB surgery and plasma insulin on postprandial VLDL kinetics. After the surgery, VLDL_1_ apoB and TG production rates were significantly decreased, whereas VLDL_2_ apoB and TG production rates remained unchanged. The TG catabolic rate was increased in both VLDL_1_ and VLDL_2_ fractions, but only the VLDL_2_ apoB catabolic rate tended to increase. Furthermore, postsurgery VLDL_1_ apoB and TG production rates, but not those of VLDL_2_, were positively correlated with insulin resistance. Insulin-mediated stimulation of peripheral lipoprotein lipolysis was also improved after the surgery. In summary, RYGB resulted in reduced hepatic VLDL_1_ production that correlated with reduced insulin resistance, elevated VLDL_2_ clearance, and improved insulin sensitivity in lipoprotein lipolysis pathways.

## Graphical abstract

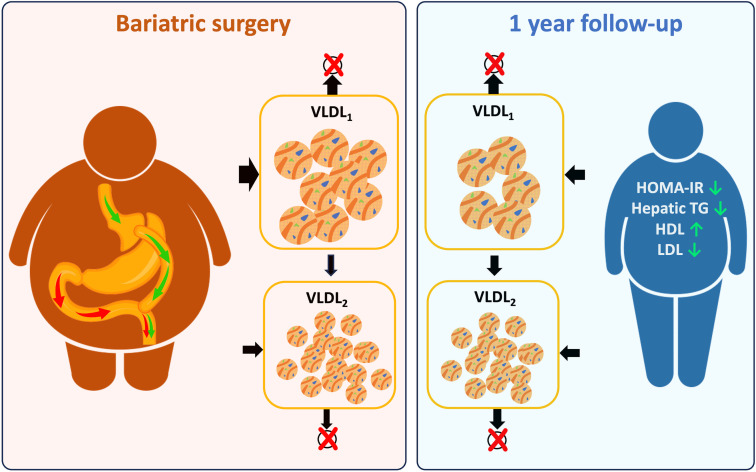

## Introduction

The prevalence of obesity has increased alarmingly over the past decades (1). Its associations with cardiovascular disease (CVD) and insulin resistance make obesity a major public health problem. Increased fasting and postprandial plasma triglyceride (TG) concentrations, reduced HDL cholesterol levels, and an increased number of LDL particles are the key characteristics of the dyslipidemia observed in patients with obesity. These lipid abnormalities result from an imbalanced lipoprotein metabolism caused by increased TG-rich lipoprotein (TRL) production and reduced TRL clearance (2, 3). Moreover, the interplay between lipid abnormalities and insulin resistance gives rise to a significantly elevated risk of developing CVD and of premature death in patients with obesity (4–6).

The pathophysiology of dyslipidemia in obesity is attributed to the hepatic overproduction and secretion of apolipoprotein B–containing (apoB-containing) TRLs and their impaired clearance from the circulation (2, 7–9). TRLs transport hydrophobic TG and cholesterol from the intestine and liver to peripheral tissues. The intestine produces and secretes chylomicrons (CMs), whereas liver produces and secretes VLDLs. VLDLs are categorized into 2 subfractions based on their sizes: VLDL_1_ and VLDL_2_, where VLDL_1_ is the larger and transports a greater amount of TG per particle. Although, other than their sizes and TG load, there is virtually no difference between VLDL_1_ and VLDL_2_, these VLDL subtypes exhibit different profiles under certain metabolic conditions. In patients with insulin resistance and/or hepatic steatosis, there is VLDL_1_, but not VLDL_2_, overproduction (10, 11). Moreover, hyperinsulinemia suppresses hepatic production of VLDL_1_ but not VLDL_2_ in both insulin-sensitive and insulin-resistant humans with low liver fat (12–14).

In target tissue capillaries, TGs in TRLs are hydrolyzed to glycerol and free fatty acids (FFAs) by lipoprotein lipase (LPL), and the products are taken up by tissue cells. As a result, circulating TRLs are transformed into smaller and denser, cholesterol-enriched lipoprotein particles. Because these small lipoprotein particles are enriched in cholesterol, they are highly proatherogenic, and their accumulation in plasma poses a significant risk for development of CVD (15). Many studies have shown a close association between dyslipidemia and the occurrence of insulin resistance and type 2 diabetes (16–19). Many hypotheses have been proposed in the literature to explain this relationship (20), but the molecular mechanism explaining how dyslipidemia, per se, may induce insulin resistance is not fully understood; more importantly, the chicken-or-egg question has not yet been answered.

Bariatric surgery is an effective treatment for patients with severe obesity; it induces sustained weight loss and improves both overall health and quality of life (21, 22). In addition to weight loss, bariatric surgery improves blood glucose regulation, insulin sensitivity, plasma lipid profiles, and BP (23, 24). Because of its high success rate, Roux-en-Y gastric bypass (RYGB) surgery is 1 of the most commonly performed bariatric procedures (25). Notably, RYGB results in dramatic improvements in insulin resistance and dyslipidemia that are not necessarily related to the extent of the weight loss (26–28). Such weight loss–independent effects of bariatric surgery often occur within a few days to weeks after the surgery and may be related to (acute) changes in caloric intake, the microbiome, and/or gastrointestinal hormones (27, 29). However, the concurrent changes that take place after the surgery make it difficult to unravel the line of events that ultimately leads to improved glucose and lipoprotein metabolism.

Computational modeling has proven to be effective for analyzing complex biological systems data and understanding hidden cause-and-effect relations (30, 31). In this regard, several computational models of human lipoprotein metabolism have been developed to analyze stable isotope–labeled tracer data (32–34). In human lipoprotein metabolism studies, tracer techniques usually are used under steady-state (fasting) conditions, where the influx and outflux of the trace are equal and all regulatory elements are assumed to be fixed (35). However, humans spend the majority of their time under nonsteady-state (postprandial) conditions. Moreover, postprandial metabolism is more strongly related to metabolic and cardiovascular disorders (36, 37). The tracer methodology and computational modeling have been used to study the nonsteady-state kinetics of simpler metabolic networks, including plasma FFAs (38) and glucose metabolism (39). However, current modeling approaches fail in using isotope-labeled tracer data to accurately describe the dynamics of human lipoprotein metabolism under postprandial conditions without using unnatural feeding regimens, such as continuous feeding of small amounts during kinetics studies (40, 41). These methods can give valuable insights, but they do not reflect the natural eating habits of humans and they also do not comprehensively describe hepatic and intestinal lipoprotein dynamics.

Recently, a physiologically relevant computational model was developed to describe the nonsteady-state dynamics of the hepatic and intestinal lipoprotein metabolism on the basis of stable isotope–labeled tracer data collected during a mixed-meal test (42). Although this model provides valuable insights about the postprandial kinetics of hepatic and intestinal lipoproteins, it does not include a physiologically relevant gastrointestinal module. Furthermore, this model does not account for the postprandial effects of insulin on lipoprotein metabolism. Therefore, it cannot be used to investigate the effects of the anatomical changes caused by bariatric surgery and/or insulin-mediated processes on lipoprotein metabolism.

In the present study, we use a detailed, multicompartmental model of hepatic and intestinal lipoprotein metabolism to investigate the postprandial interactions between glucose and lipid pathways in patients with severe obesity and after bariatric surgery–induced weight loss. We recruited 24 patients and performed detailed postprandial lipoprotein kinetics experiments at baseline and at 1 year after RYGB. We also performed hyperinsulinemic-euglycemic clamps (HECs) to assess the interactions between lipoprotein and glucose–insulin kinetics. Taken together, our model provides deep insight into the postprandial lipoprotein metabolism of patients with severe obesity and into the changes induced by bariatric surgery.

## Results

### Patient characteristics.

The study population comprised 24 patients with severe obesity (Table 1). At 1-year follow-up, RYGB resulted in significant weight loss in all patients. In addition, RYGB was associated with important improvements in metabolic health, including reduced intrahepatic TG (IHTG) content, plasma lipid concentrations, insulin sensitivity, and glycemia (Table 1).

### Postprandial concentrations of VLDL_1_ TG and apoB, and VLDL_2_ TG are reduced after RYGB.

**Figure 1:**
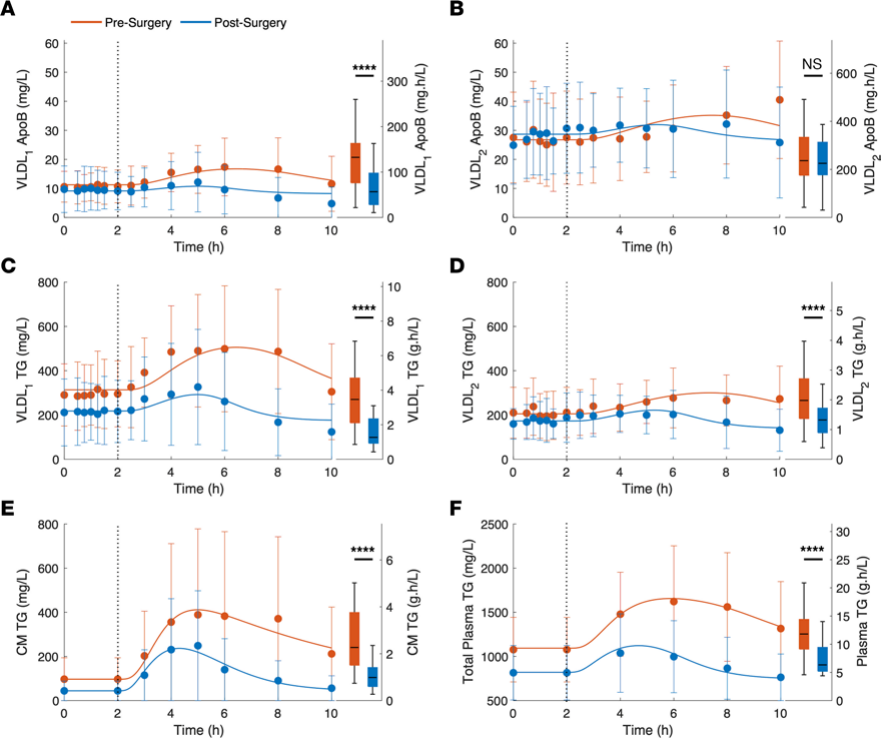
Plasma apoB and TG concentration time courses. (**A**–**F**) Presurgery (red) and postsurgery (blue) plasma VLDL_1_ and VLDL_2_ apoB (**A** and **B**), TG (**C** and **D**), plasma CM TG (**E**), and plasma TG (**F**) concentrations measured during the lipoprotein kinetics studies. Filled circles and error bars show data as mean ± SD. Solid curves show model simulations generated with model parameters estimated from average data (Supplemental Table 2). Dotted vertical lines mark mixed-meal ingestion time points. The box plots show the presurgery (red) and postsurgery (blue) AUCs calculated for the postprandial state (2–10 hours). Statistical significance was tested with a paired-sample *t* test. ****P < 0.0005.

All patients underwent a lipoprotein kinetics study that included a mixed-meal test at baseline and 1 year after the surgery. During the study, plasma VLDL_1_ and VLDL_2_ apoB (Figure 1, A and B), TG (Figure 1, C and D), CM TG (Figure 1E), and total plasma TG (Figure 1F) were measured up to 10 hours. At 2 hours, patients were asked to consume a mixed meal, and the postprandial phase started. In Figure 1, box plots show the presurgery (red) and postsurgery (blue) AUCs calculated for the postprandial state. One of the key differences between pre- and postsurgery apoB and TG profiles was the response to the mixed meal. Before surgery, meal ingestion was followed by pronounced VLDL_1_ and VLDL_2_ apoB (Figure 1, A and B, red) and TG (Figure 1, C and D, red) accumulation in the plasma. However, after the surgery, VLDL_1_ and VLDL_2_ apoB (Figure 1, A and B, blue) and TG (Figure 1, C and D, blue) concentrations were only moderately increased after the meal. After the surgery, the VLDL_1_ apoB AUC was significantly reduced (Figure 1A), whereas VLDL_2_ apoB AUC remained unchanged (Figure 1B). On the other hand, both VLDL_1_ and VLDL_2_ TG AUCs were significantly reduced after the surgery (Figure 1, C and D). The average TG to apoB ratio, which is calculated as the ratio of the TG to apoB AUCs, did not change in the VLDL_1_ fraction (31.1 ± 7.1 vs. 28.8 ± 8.2 mg TG/mg apoB), but it was reduced in the VLDL_2_ fraction (8.7 ± 2.3 vs. 6.3 ± 1.9 mg TG/mg apoB; *P* < 0.001) after the surgery. This indicates a decline in average VLDL_2_ particle size after the surgery. The CM TG AUC (Figure 1E) and plasma TG AUC (Figure 1F) were also significantly reduced after the surgery. Our modeling results show that postsurgery reduction in the postprandial CM TG concentration resulted from reduced lipid absorption from intestine and enhanced CM TG clearance rate. After the surgery, the estimated CM TG clearance rate was increased by 72% on average (*P* < 0.001).

Figure 1 also shows that after the surgery, postprandial plasma VLDL apoB and TG exhibit faster dynamics. After the surgery, plasma VLDL_1_ and VLDL_2_ apoB and TG levels peaked earlier, compared with presurgery. After the surgery, postprandial plasma VLDL_1_ apoB peak time was decreased from 4.8 ± 1.5 to 3.3 ± 0.7 hours (*P* < 0.0005), and plasma VLDL_2_ apoB peak time was reduced from 6.0 ± 1.6 to 4.1 ± 1.2 hours (*P* < 0.0001). Similar peak-time reductions were observed for plasma VLDL_1_ TG (4.8 ± 1.5 vs. 3.3 ± 0.6 hours; *P* < 0.0005) and VLDL_2_ TG (5.7 ± 1.6 vs. 3.7 ± 0.8 hours; *P* < 0.0001) after the surgery. Moreover, postsurgery plasma VLDL_1_ and VLDL_2_ apoB and TG concentrations returned to their respective baselines within the observation time frame; this was not the case before the surgery.

A similar pattern was evident for CM TG and plasma TG time courses. Before the surgery, meal intake was followed by a significant increase in CM TG (Figure 1E, red) and plasma TG (Figure 1F, red) concentrations. After the surgery, postprandial CM TG and plasma TG elevations were lower as compared with presurgery status (Figure 1, E and F, blue). Our results show that after the surgery, CM TG peak time does not change much (4.9 ± 0.4 vs. 4.6 ± 0.6 h). The lack of reduction in CM TG peak time may be due, in part, to the slower meal consumption after the surgery (10 vs. 30 minutes; see Methods and Supplemental Methods for details). However, total plasma TG peak time is reduced after the surgery (5.9 ± 0.9 vs. 5.1 ± 0.8 h; *P* < 0.01). After surgery, CM TG and plasma TG levels also returned to their baselines within the observation time frame (Figure 1, E and F, blue).

### RYGB is associated with reduced postprandial VLDL_1_ TG and apoB production and increased postprandial VLDL_2_ TG clearance.

**Figure 2:**
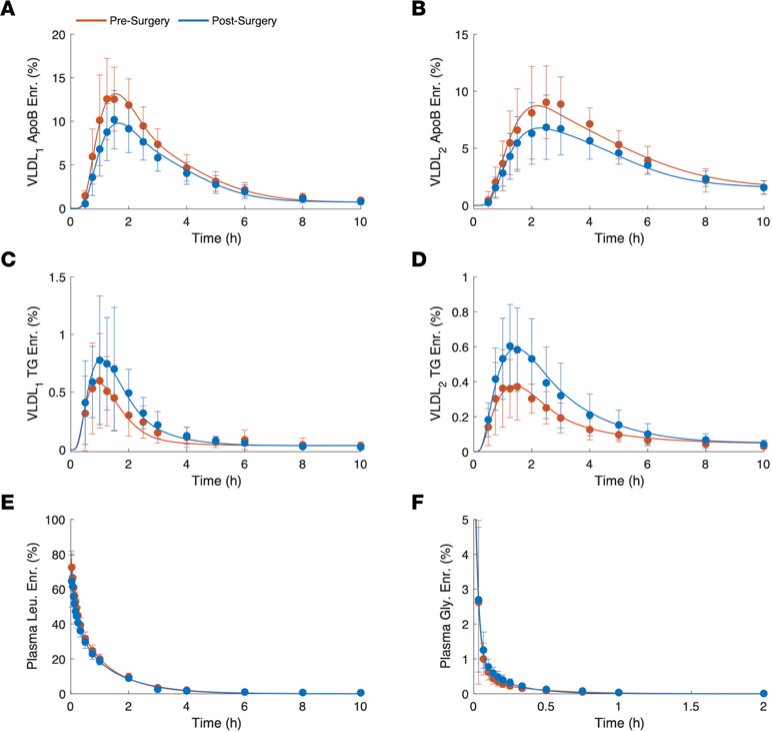
Leucine and glycerol enrichment time-courses. Presurgery (red) and postsurgery (blue) population averages for enrichment data (filled circles with error bars) and the model simulations (solid lines). (**A** and **B**) VLDL_1_ and VLDL_2_ apoB [5,5,5-^2^H_3_]-leucine (Leu.) enrichments (Enr.). (**C** and **D**) VLDL_1_ and VLDL_2_ TG [1,1,2,3,3-^2^H_5_]-glycerol (Gly.) enrichments. (**E**) Plasma [5,5,5-^2^H_3_]-leucine enrichment. (**F**) Plasma [1,1,2,3,3-^2^H_5_]-glycerol enrichment. Data are presented as mean ± SD.

To gain more insight into the mechanisms underlying the observed changes in lipoprotein kinetics, isotopic enrichment data from different pools were assessed and analyzed using the computational model described in Methods. In Figure 2, pre- and postsurgery isotopic enrichment data from VLDL_1_ and VLDL_2_ apoB (Figure 2, A and B) and TG (Figure 2, C and D) pools, as well as plasma leucine (Figure 2E) and glycerol (Figure 2F) pools, are shown along with the model simulations. The results in Figures 1 and 2 show that the dynamics of the plasma concentrations and enriched materials in different pools are accurately captured by the computational model, allowing the calculation of the parameters that describe VLDL_1_ and VLDL_2_ apoB and TG kinetics in detail. Pre- and postsurgery kinetic parameters were estimated for each patient, and their averages are given in Table 2.

After the surgery, VLDL_1_ apoB production rate was decreased (Table 2), whereas VLDL_2_ apoB direct production rate, which is the rate at which apoB is directly secreted in the form of VLDL_2_ from liver, did not change. As a consequence, the relative fraction of apoB that was directly secreted as VLDL_2_ from liver was increased from 32% ± 14% to 43% ± 16% (*P* <0.05) after the surgery. On the other hand, the fractional transfer rate (FTR) of apoB from the VLDL_1_ pool to the VLDL_2_ pool tended to increase. Consequently, the total VLDL_2_ apoB production rate, which is the sum of the VLDL_2_ apoB that is directly secreted from liver and the VLDL_2_ apoB that is derived from the VLDL_1_ pool in circulation, is slightly increased, but this change did not reach statistical significance. The VLDL_1_ TG production rate was significantly reduced, as was the VLDL_2_ TG direct production rate. These findings indicate a noticeable shift in the distribution of hepatic TG secretion toward VLDL_2_ (18% ± 12% vs. 23% ± 11%; *P* < 0.1) after the surgery. Together with the increase observed in the VLDL_1_ TG FTR, the total VLDL_2_ TG production rate remained the same after surgery.

After surgery, the VLDL_1_ apoB fractional catabolic rate (FCR) did not change (Table 2), whereas the VLDL_2_ apoB FCR tended to increase. In line, the VLDL_1_ apoB fractional direct catabolic rate (FDC), which is the rate at which VLDL_1_ apoB is directly removed from the circulation, did not change. After the surgery, a remarkable increase took place in VLDL TG catabolic rates, whereby VLDL_1_ TG FCR tended to increase from 27.7 ± 23.4 to 34.0 ± 17.0 pools/d (*P* = 0.1) and VLDL_2_ TG FCR was significantly increased from 10.8 ± 6.1 to 17.0 ± 10.1 pools/d (*P* = 0.002). However, the VLDL_1_ TG FDC did not change after surgery.

### Insulin-mediated stimulation of lipoprotein lipolysis is enhanced after surgery.

**Figure 3:**
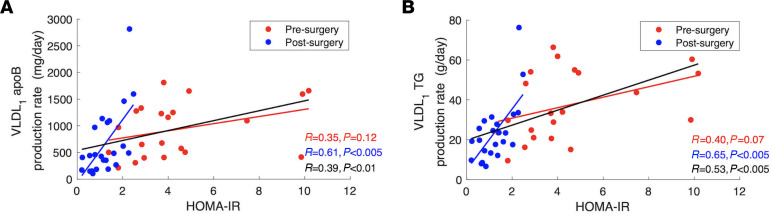
Relationship between pre- and postsurgery VLDL_1_ apoB and TG production rates and HOMA-IR. (**A** and **B**) The regression lines for pre- and postsurgery data and combined data are shown in red, blue, and black, respectively. Correlation coefficients (*r*) and associated *P* values are reported in each panel.

After surgery, the homeostatic model assessment of insulin resistance (HOMA-IR) was significantly reduced (4.4 ± 2.5 vs. 1.2 ± 0.7; *P* < 0.005), and it was positively related to VLDL_1_ apoB and TG production. After the surgery, there was a significant positive correlation between HOMA-IR and VLDL_1_ apoB (*r* = 0.61; *P* < 0.005; Figure 3A, blue) and TG (*r* = 0.65; *P* <0.005; Figure 3B, blue) production rates. In the presurgery condition, trends between VLDL_1_ apoB and TG production and HOMA-IR were the same, but the correlations were not statistically significant. When pre- and postsurgery data were combined and analyzed together, the correlations between HOMA-IR and VLDL_1_ apoB production (*r* = 0.39; *P* < 0.01; Figure 3A, black) and VLDL_1_ TG production (*r* = 0.53; *P* < 0.005; Figure 3B, black) remained significant. On the other hand, VLDL_2_ apoB or TG production was not correlated with HOMA-IR before or after surgery.

**Figure 4:**
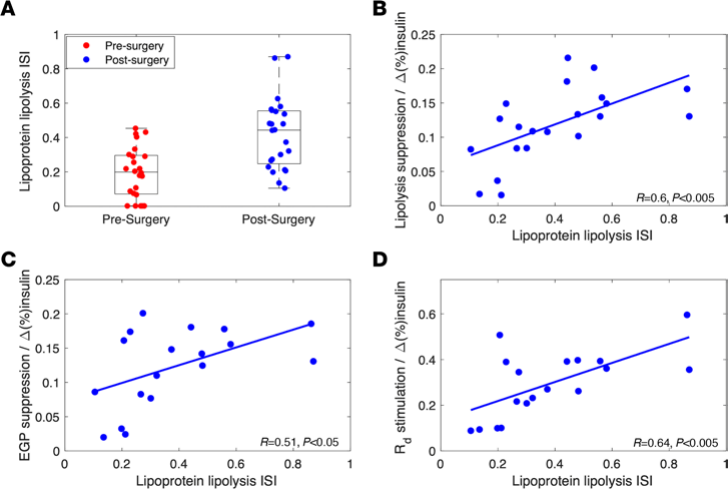
Relationship between lipoprotein lipolysis ISI and tissue specific insulin responsiveness indices. (**A**) Pre- and postsurgery lipoprotein lipolysis ISI. (**B**) Postsurgery insulin-mediated peripheral lipolysis suppression per insulin increased over basal level (Δ[%]insulin) from the clamp studies vs lipoprotein lipolysis ISI. (**C**) Postsurgery insulin-mediated EGP suppression per Δ(%)insulin versus lipoprotein lipolysis ISI. (**D**) Postsurgery insulin-mediated R_d_ stimulation per Δ(%)insulin versus lipolysis ISI. In each panel, the regression lines for the postsurgery data are shown in blue. Correlation coefficients (*r*) and associated *P* values are reported in each panel.

Because insulin regulates both glucose and lipid homeostasis, we aimed to quantify the contribution of insulin to the regulation of postprandial lipoprotein metabolism, by incorporating an insulin-mediated stimulation of lipoprotein lipolysis pathway into the computational model. The responsiveness of the lipoprotein lipolysis pathway to circulating insulin can be expressed as the lipoprotein lipolysis insulin sensitivity index (ISI), which was estimated by model from the individual patient experimental data as described in the Supplemental Methods. In 5 patients, the model detected no insulin effect on lipoprotein lipolysis before surgery, but there was a detectable effect at 1-year follow-up. In all patients, the postsurgery lipoprotein lipolysis ISI was significantly increased (0.2 ± 0.15 vs. 0.42 ± 0.21; *P* < 0.001; Figure 4A). Furthermore, parameters of insulin sensitivity from the clamp studies were directly correlated to the model-derived lipolysis ISI in patients after surgery (Figure 4, B–D).

## Discussion

Data from this study demonstrate that bariatric surgery is not only associated with significant weight loss and improved metabolic health but also with decreased postprandial VLDL_1_ production, increased postprandial VLDL_2_ clearance, and improved insulin sensitivity of the lipolysis pathway. Using physiology-based kinetic modeling, we provide deeper insight into the complexity of human lipoprotein homeostasis. We show that the reduction in plasma VLDL apoB after bariatric surgery is a consequence of seemingly opposing effects on VLDL_1_ versus VLDL_2_ apoB kinetics: VLDL_1_ apoB production was reduced, with an unaltered VLDL_1_ apoB catabolic rate, whereas the VLDL_2_ apoB direct production rate was unaltered, but its clearance rate tended to be increased. Together, these findings imply a shift from the hepatic secretion of large VLDL_1_ particles before surgery toward the secretion of smaller VLDL_2_ particles after surgery. Consistent with such a shift, we found that the relative fraction of hepatic apoB that was secreted in the form of VLDL_2_ increased by more than 33% after surgery. Moreover, bariatric surgery–induced weight loss was associated with decreased VLDL TG concentrations in both fractions, and our computational modeling indicated that this reduction was due to decreased VLDL_1_ TG production as well as increased VLDL_1_ and VLDL_2_ TG turnover.

Previously, Padilla et al. (41) studied the impact of gastric bypass and sleeve gastrectomy on hepatic apoB kinetics under constant feeding conditions. They reported a significant decrease in plasma VLDL apoB after both gastric bypass and sleeve gastrectomy surgeries, due to reduced hepatic production and increased fractional catabolic rate. For the gastric bypass, their report showed a trend toward a decrease in hepatic apoB production, but the apoB fractional catabolic rate remained the same (41). However, their study did not account for the different VLDL subfractions and, more importantly, their data were collected under a constant feeding regimen, which does not reflect natural eating habits. Several other studies investigated postprandial plasma lipid profiles after bariatric surgery. In patients with obesity and/or type 2 diabetes, it was shown that postprandial plasma TG and plasma cholesterol concentrations were significantly reduced during a mixed-meal test that was performed 2 weeks after the sleeve gastrectomy or gastric bypass (43). In a follow-up study, similar postprandial reductions in plasma TG and cholesterol concentrations were reported 2 years after the surgery (44). In a third study, a standard oral fat-load test was performed 3 months after sleeve gastrectomy, and a significant reduction in postprandial VLDL CM-remnant TG concentration was reported (45). Although, the reported postsurgery changes in plasma TG concentrations in these studies are consistent with our findings, the design of these studies did not allow the researchers to investigate the production or the turnover kinetics of lipoproteins. Hence, they could not provide mechanistic insights of reduced plasma concentrations.

An advantage of our computational modeling–based approach was that the detailed insight could be obtained in pre- and postsurgery dynamic regulation of lipoprotein metabolism during the postprandial state. Our data show that in the presurgery phase, there is a profound VLDL accumulation in plasma during the postprandial state. In line with earlier reports, we hypothesize that this is primarily due to the competition for LPL-mediated lipoprotein lipolysis (46–50). The competition between different lipoprotein species for lipolysis pathways becomes particularly evident in the postprandial state as the digested lipids enter the circulation in the form of CMs. Studies show a positive relation between particle size and the lipolysis rate (51–53), which gives CMs the priority for lipolysis by LPL. Hence, in the postprandial state, increased CM size in the circulation promotes competition and results in a significant reduction in VLDL apoB and TG FCRs and FTRs. Our results show that, after surgery, postprandial plasma VLDL apoB and TG concentrations do not increase much (Figure 1, blue), compared with the presurgery condition (Figure 1, red), for 3 reasons. First, after surgery, hepatic apoB and TG production are significantly reduced. Second, reduced postsurgery intestinal lipid absorption and increased CM TG clearance rate result in lower plasma CM TG (Figure 1E, blue). Although, reduced intestinal lipid absorption is a model estimate and has not been verified by measuring lipids in stool samples, it is consistent with previous studies showing a significant reduction in intestinal lipid absorption after RYGB surgery (54–58). Hence, in the postprandial state, the competition between hepatic and intestinal lipoproteins for the lipolysis pathways remains weak. Third, after surgery, postprandial stimulation of lipoprotein lipolysis by insulin is greatly improved (Figure 4A). As a consequence of these factors, after surgery, postprandial VLDL apoB and TG accumulation in plasma remains modest. Furthermore, elevated plasma TG and apoB concentrations return to their baselines within the time course of the study (8 hours), whereas postprandial lipids remain elevated for more than 8 hours, on average, in the presurgery condition. This implies that, after surgery, after consuming a meal, the plasma lipid profiles are more likely to return to their baselines before the next meal is consumed and this effectively prevents residual lipids from the previous meal to further increase plasma lipid concentrations.

In this study, we also demonstrated significant improvements in postsurgery insulin-responsiveness indices, and a positive correlation between insulin resistance and hepatic VLDL_1_ production after the surgery. Insulin reduces hepatic VLDL secretion by reducing apoB lipidation and promoting apoB degradation in the hepatocyte (59–61). The HOMA-IR index was significantly reduced after surgery. We showed that HOMA-IR was positively correlated with postsurgery VLDL_1_ TG and apoB production (Figure 3, A and B) but not with VLDL_2_ apoB or TG production. This finding emphasizes the role that insulin plays in the regulation of hepatic lipoprotein production, whereby patients with high insulin resistance tend to produce greater amounts of large hepatic lipoproteins. The lack of association between VLDL_2_ apoB or TG production and HOMA-IR might be explained by independent regulation of hepatic VLDL_1_ and VLDL_2_ apoB production, as suggested before (10, 11). This indicates that the production of larger hepatic lipoprotein particles is increased with insulin resistance, but the production of smaller lipoprotein particles is not affected. The lack of a statistically significant association between presurgery insulin resistance indices and estimated kinetic parameters might be due to the relatively high insulin resistance and large interpatient variability in comparison with the relatively small population size. Nevertheless, when pre- and postsurgery data were analyzed together, the correlations between HOMA-IR and VLDL_1_ apoB and TG production (Figure 3, A and B, black) remained significant. This may indicate that, after surgery, the associations between HOMA-IR and VLDL_1_ apoB and TG production do not change but become more pronounced.

We also show in this study a postsurgery improvement in insulin-mediated stimulation of the lipoprotein lipolysis pathway. Insulin is known to stimulate lipoprotein lipolysis by its impact on LPL at both the transcriptional and posttranslational levels (62, 63). To quantify the responsiveness of the lipoprotein lipolysis pathway to circulating insulin, we have introduced the lipoprotein lipolysis ISI, which was estimated for each patient by using individual experimental data with the model. Our results, indeed, show that insulin-mediated stimulation of lipoprotein lipolysis was improved after the surgery, as reflected in the significantly increased lipoprotein lipolysis ISI (0.20 ± 0.15 vs. 0.42 ± 0.20; *P* < 0.001; Figure 4A). Moreover, calculated postsurgery lipoprotein lipolysis ISI values were strongly correlated with measured insulin-mediated adipose tissue lipolysis suppression (Figure 4B), endogenous glucose production (EGP) suppression (Figure 4C), and glucose disposal rate (R_d_) stimulation (Figure 4D) indices. because the lipoprotein lipolysis ISI is defined as the sensitivity of the lipoprotein lipolysis pathway to the fractional increase in insulin over the baseline, we compared lipoprotein lipolysis ISI with the clamp-derived sensitivity indices normalized over the fractional increase in insulin during the clamp studies. However, the associations between lipoprotein lipolysis ISI and clamp-derived insulin sensitivity parameters were not present for presurgery data (Supplemental Figure 5; supplemental material available online with this article; https://doi.org/10.1172/jci.insight.166905DS1). This may be due, in part, to the fact that model could not detect an insulin-mediated lipoprotein lipolysis stimulation in the data of 5 of the 24 patients before surgery. Nevertheless, a linear trend between lipoprotein lipolysis ISI and insulin-mediated lipolysis suppression was evident in the combined pre- and postsurgery data (Supplemental Figure 5B), which was not the case for other clamp-derived insulin-sensitivity parameters (Supplemental Figure 5, C and D). The dynamics of the insulin-mediated suppression of the hepatic apoB production was also incorporated into the model as a delayed forcing signal generated by the portal vein insulin; the portal vein insulin signal was derived from plasma insulin data. However, insulin-mediated effects on the hepatic apoB production pathway remained undetectable due to the uncertainty associated with portal vein insulin concentration and resulting parameter estimates. Therefore, insulin-mediated suppression of the hepatic apoB production was removed from the final version of the model.

Glucagon-like peptide-1 (GLP-1) is best known for its role in glucose homeostasis and insulinotropic effects (64–66). However, GLP-1 also plays a direct role in lipid and lipoprotein metabolism (67), and thus the GLP-1 receptor pathway has been the focus of pharmacological lipid research (68). GLP-1 reduces intestinal CM production and secretion (69, 70), and activation of GLP-1 receptors reduces hepatic VLDL production (71, 72). Moreover, GLP-1 also triggers a signal through the intrinsic gut–liver axis and ameliorates diet-induced hepatic VLDL overproduction (73). These studies suggest that, other than being a potent insulin secretagogue, GLP-1 regulates lipoprotein metabolism in an insulin-independent manner. Our results show that, after RYGB surgery, fasting GLP-1 levels do not change (3.5 ± 3.9 vs. 4.1 ± 3.6 pmol/L; *P* = 0.26; Supplemental Figure 4). However, postprandial GLP-1 levels are significantly increased (AUC: 2139.2 ± 1093.5 vs. 7204.3 ± 4106.4 pmol/min/L; *P* <0.005; Supplemental Figure 4), which is consistent with earlier reports showing a significant increase in GLP-1 levels after RYGB surgery (74–79). It is suggested that elevated GLP-1 levels play an important role in several metabolic improvements and diabetes remission after RYGB surgery (27, 78–81). Thus, postsurgery reduction in VLDL production and improved insulin-mediated processes may be, in part, a consequence of elevated GLP-1 levels.

TG homeostasis directly influences hepatic steatosis, for which our data indicate a significant reduction in IHTG after surgery (9.9% ± 9.0% vs. 4.0% ± 1.7%; *P* < 0.05) as reported before (82–84). Adiels et al. (10) proposed that increased hepatic fat content as a consequence of increased FFA flux to the liver due to insulin resistance results in overproduction of larger VLDL_1_ particles. However, it is not clear whether this association was a direct impact of hepatic insulin resistance or increased IHTG that was secondary to insulin resistance, because insulin resistance and hepatic steatosis are common comorbidities (85, 86). Indeed, we showed a strong association between postsurgery HOMA-IR and VLDL_1_ apoB and TG production but not VLDL_2_, which is consistent with earlier reports (10, 11).

We used a physiologically based, large computational model and a comprehensive data set to investigate the impact of RYGB surgery on VLDL_1_ and VLDL_2_ apoB and TG kinetics under the nonsteady-state postprandial condition during a mixed-meal test. The study was designed to capture dynamic responses to natural eating regimens as closely as possible. The complex and dynamic nature of lipoprotein metabolism, together with multiple interactions occurring postprandially, made it necessary to make assumptions and simplifications during the model development. We acknowledge that like all models that have been proposed in the literature and all the models that will follow, our model is not a complete account of the entire physiological processes involved. The developed model is not intended to capture all the biochemical or molecular details of the lipoprotein metabolism. The computational model was developed in a way to extract as much information as possible from the available data while preserving physiological relevance.

### Conclusion.

We conclude that physiologically based mathematical modeling of postprandial apoB and TG metabolism in different VLDL fractions in combination with gold standard measurements of insulin sensitivity provide deep insight into the effects of RYGB surgery on lipid handling and its interaction with glucose metabolism and insulin in the postprandial state. RYGB restores the homeostatic balance between insulin sensitivity and TG production and catabolism. Taken together, our experimental data, combined with computational modeling, show that RYGB in morbidly obese patients results in reduced postprandial VLDL TG due to reduced VLDL_1_ production and increased VLDL_2_ TG clearance rates, with improved responsiveness of lipoprotein homeostasis to circulating insulin levels.

## Methods

### Design.

This multicenter, observational intervention study was part of RESOLVE (A systems biology approach to RESOLVE the molecular pathology of two hallmarks of patients with metabolic syndrome and its comorbidities; hypertriglyceridemia and low HDL-cholesterol), a European research program on the metabolic syndrome. We designed the present study to evaluate postprandial lipoprotein kinetics in humans before and after bariatric surgery–induced weight loss and their relation to insulin-mediated processes. For this purpose, we developed a physiologically based computational model of human lipoprotein metabolism and used this model to analyze in vivo data collected during the baseline (before surgery) and 1 year after the RYGB.

### Study population.

We recruited patients with severe obesity from the outpatient clinic of 2 obesity centers in the Amsterdam metropolitan area. Patients were eligible to participate in the present study if they (a) were older than 18 years; (b) met criteria for bariatric surgery in accordance with national guidelines (87); (c) were scheduled for elective RYGB; and (d) had stable weight for at least 3 months before surgery. Exclusion criteria were (a) the use of alcohol (>2 units/d) or recreational drugs; (b) the use of lipid-lowering drugs, exogenous insulin, incretin mimetics, or psychoactive medication; (c) chilDVHood-onset obesity; or (d) any somatic disorder except for common obesity-related conditions (e.g., dyslipidemia, hypertension, obstructive sleep apnea).

### Lipoprotein kinetics studies.

Lipoprotein kinetics experiments were performed using [5,5,5-^2^H_3_]-leucine and [1,1,2,3,3-^2^H_5_]-glycerol to determine in vivo apoB and TG fluxes in VLDL_1_ and VLDL_2_ fractions (Supplemental Figure 1A). Experiments were performed shortly (<4 weeks) prior to the scheduled RYGB surgery and repeated 1 year after the operation. After an overnight fast, [5,5,5-^2^H_3_]-leucine (7 mg/kg BW; 99% enriched; Cambridge Isotopes) and [1,1,2,3,3-^2^H_5_]-glycerol (500 mg; >99% enriched; Cambridge Isotopes) were infused via a venous catheter. Two hours after tracer infusion, patients received a liquid mixed meal that consisted of 2 bottles of Fresubin Protein Energy (Fresenius Kabi), 40 mL of olive oil, 2 g of cacao powder, and 5 tablets of a noncaloric sweetener (Hermesetas; Hermes Sweeteners) (Supplemental Table 1). Patients were instructed to consume the meal within 10 minutes before surgery or within 30 minutes at 1-year follow-up. After the surgery, patients were given more time to consume the meal because they experienced difficulty in completing the meal within 10 minutes. At 5, 15, 30, 45, 60, 75, 90, 120, 150, 180, 240, 300, 360, 480, 600, and 1440 minutes after infusion, venous blood samples were drawn for the determination of [5,5,5-^2^H_3_]-leucine and [1,1,2,3,3-^2^H_5_]-glycerol enrichment in plasma and lipoprotein fractions.

### Isolation of VLDL subfractions.

VLDL_1_ and VLDL_2_ fractions were isolated from plasma by 3-step gradient ultracentrifugation using a SW41 rotor (Beckman) in an Optima XPN-100 Beckman ultracentrifuge. In short, the density (d) of 4 mL of plasma was adjusted to 1.1502 g/mL with NaCl. Then 0.5 mL of NaBr/NaCl (d = 1.182 g/mL) and 4 mL of plasma (d = 1.1502 g/mL) was transferred to an ultraclear Beckman SW41 tube. The gradient was formed by layering 2 mL of salt solutions of the following densities on the top of the plasma: (a) 1.079 g/mL; (b) 1.0722 g/mL; (c) 1.0641 g/mL; and (d) 1.0588 g/mL. The different fractions were isolated using the following conditions: for CMs (d < 1.006 g/mL): 30 minutes, 260,639 g; for VLDL_1_: 51 minutes, 260,639 g; and for VLDL_2_: 16.36 hours, 55,521 g. At each step, the upper 1 mL was aspirated and replaced by the appropriate density fraction. Isolated lipoprotein fractions were frozen at –80 °C until further analysis.

### Determination of isotopic enrichments.

To determine leucine enrichment in apoB, VLDL_1_ and VLDL_2_ fractions were precipitated with isopropanol, delipidated with ethanol-diethyl ether, dried, and hydrolyzed with 6M HCl at 110°C for 24 hours. The samples were then prepared for analysis of leucine enrichment as described (88, 89). Briefly, leucine enrichment was determined on a gas chromatography–mass spectrometry (GC-MS) (GC-MSD5975c; Agilent Technologies) equipped with a VF17ms column operated in selected ion monitoring mode, using norleucine as an internal standard. To calculate isotope enrichments, the average value of the *m/z* 161:158 ratio was determined using a calibration curve with known quantities of labeled and unlabeled leucine (90). The resulting *m/z* 161:158 was expressed as molar percent excess.

To determine glycerol enrichment in TG within VLDL_1_ and VLDL_2_, the isolated fractions were precipitated with isopropanol, delipidated with alcohol/diethyl ether, and solubilized in isopropanol. The phospholipids were removed by adding 2 g of activated zeolite (Merck) to each tube. After centrifugation, the samples were evaporated under N_2_ at 80°C. Isopropanol was added and the samples were transferred to a 1.5 mL vial. The glycerol extracts were saponified with 2% KOH in ethanol, incubated for 2 hours at 60°C, and dried under N_2_. Heptafluorobutyric acid (Sigma-Aldrich) and ethyl acetate, at a 1:3 ratio, and standards and controls were added and incubated for 10 minutes at 70°C. After evaporation under N_2_, the samples were solved in ethyl acetate and analyzed by GC-MS as described before (89).

### HEC.

We determined basal and insulin-mediated glucose fluxes as well as lipolysis rates during a 2-step HEC using [6,6-^2^H_2_]-glucose and [1,1,2,3,3-^2^H_5_]-glycerol (Supplemental Figure 1B), as described elsewhere (91, 92). Briefly, primed continuous infusions of [6,6-^2^H_2_]-glucose (prime: 11 μmol/kg; continuous: 0.11 μmol/kg/min; >99% enriched; Cambridge Isotopes) and [1,1,2,3,3-^2^H_5_]-glycerol (prime: 1.6 μmol/kg; continuous: 0.11 μmol/kg/min; >99% enriched; Cambridge Isotopes) were started and continued until the end of the study. Basal glucose (EGP) and glycerol (from lipolysis) production were determined after 2 hours of tracer equilibration. Next, insulin-mediated suppression of EGP and insulin-mediated suppression of lipolysis were determined after 2 hours of low-dose insulin infusion (step 1: Actrapid 20 mU/m^2^ body surface area/min; Novo Nordisk Farma). Finally, insulin-stimulated R_d_ was determined after an additional 2 hours of high-dose insulin infusion (step 2: 60 mU/m^2^/min). During insulin infusion, blood glucose concentration was held at 5 mmol/L by frequent bedside monitoring and variable exogenous glucose infusion (enriched with 1% [6,6-^2^H_2_]-glucose to maintain stable enrichment in the plasma pool).

### Biochemical analyses.

Plasma glucose concentrations were determined with the glucose-oxidation method using a Biosen C-Line glucose analyzer (EFK Diagnostics). Insulin and cortisol were determined by immunoassay on an Immulite 2000 system (Diagnostic Products) with intra-assay variations of 4–5% and 3–6%, respectively. Glucagon was determined by radioimmunoassay (Linco Research) with an intra-assay variation of 4–8%. Plasma FFAs were analyzed by enzymatic colorimetric assay (NEFA-C kit, Wako Chemicals). Plasma total cholesterol, LDL cholesterol, HDL cholesterol, and TG were analyzed by a Selektra autoanalyzer (Sopachem). Plasma apoB was determined by immunoturbidimetric assay (Wako Chemicals) by a Selektra autoanalyzer.

### Calculations of basal and insulin-mediated fluxes.

We calculated glucose fluxes during HEC (EGP and the R_d_) using modified versions of Steele equations for the steady state (basal fluxes) or nonsteady state (fluxes during the insulin infusion) (93, 94). Hepatic insulin sensitivity was defined as the percent suppression of EGP by step-1 hyperinsulinemia; peripheral or muscle insulin sensitivity was defined as the percent stimulation of glucose R_d_ by step-2 hyperinsulinemia (95). Basal whole-body lipolysis was defined as the glycerol rate of appearance, which, in turn, was determined using the tracer dilution method (96). Adipose tissue insulin sensitivity was defined as the percent suppression of lipolysis by step-1 hyperinsulinemia (97). Finally, given the interindividual variation in insulin clearance, parameters of insulin sensitivity were normalized to insulin levels during the clamp.

### Determination of liver fat content and excess BW.

IHTG was determined by proton magnetic resonance spectroscopy as described before (98). Excess weight was calculated as the weight that corresponds to the difference between the patient’s BMI and a cutoff BMI value of 25 kg/m^2^.

### Computational modeling.

We used a computational modeling approach to investigate the effects of the RYGB surgery on postprandial lipoprotein kinetics and to explore the complex interactions between glucose and lipid fluxes. To achieve this, we translated the metabolic network of systemic lipoprotein metabolism into a physiologically based mathematical model, as illustrated in Supplemental Figure 2. The model describes systemic lipoprotein kinetics using 5 interconnected modules: gastrointestinal, plasma, liver, tracer injection, and insulin. For computational simulations and analyses, the system dynamics were described with a system of ordinary differential equations. The mathematical model was then implemented into the MATLAB programming environment (MathWorks; R2018b). The kinetic transfer rate parameters were estimated from the experimental isotopic enrichment and biochemical concentration data, using MATLAB’s optimization toolbox. The details of the computational model are given in Supplemental Methods.

### Statistics.

All statistical analyses were performed using the MATLAB programming environment. Normally distributed data are presented as mean ± SD. We used median and IQR to present nonnormally distributed data. We used 1-tailed, paired-sample *t* tests to compare baseline data with 1-year follow-up data. Bivariate correlations were evaluated using Pearson correlation coefficients. Findings were considered significant if *P* < 0.05.

### Study approval.

The study was approved by the Amsterdam University Medical Center Medical Ethics Committee. All participants provided written informed consent in accordance with the Declaration of Helsinki. The study was prospectively registered in the Netherlands Trial Registry (identifier NL4531; www.trialregister.nl).

### Data availability.

Computer codes for the computational model and data files are publicly available in the GitHub data repository at https://github.com/vehpi/lipoprotein_kinetics/tree/27f0e42919d638e988a3a71d7cdb570e6cb20fdb Additional information will be provided by the corresponding author upon request; values for all data points in graphs are reported in the Supporting Data Values file.

## Author contributions

GMDT, NAWVR, MJS, AKG, and MN designed the study; KWTH, PWG, and MJS performed the human studies; DVH, HS, HJ, and AWMS performed the laboratory analysis; VY and NAWVR developed the computational model; VY wrote the computer programs, created figures and tables, and wrote the first draft; VY, KWTH, GMDT, NAWVR, MJS, AKG, and MN revised the first draft and finalized the manuscript.

## Supplementary Material

Supplemental data

Supporting data values

## Figures and Tables

**Table 1:**
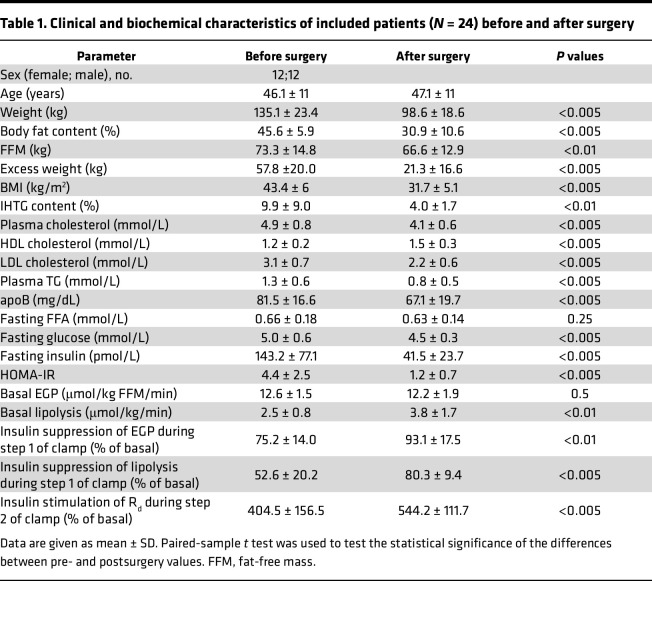
Clinical and biochemical characteristics of included patients (*N* = 24) before and after surgery

**Table 2:**
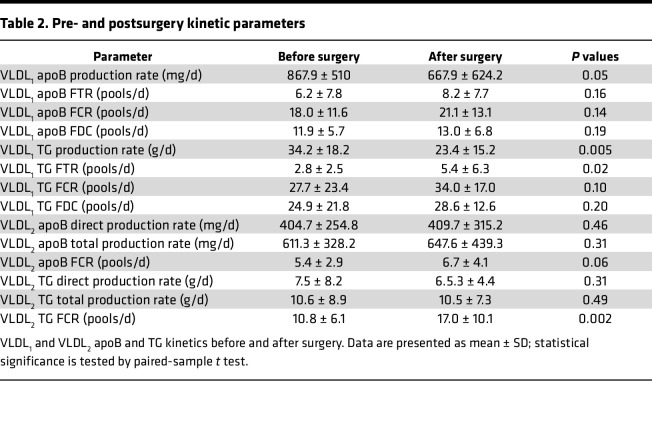
Pre- and postsurgery kinetic parameters

## Version 1 07/11/2023

In-Press Preview

## Version 2 08/22/2023

Electronic publication
