# Supplementary material for: Bariatric surgery improves postprandial VLDL kinetics and restores insulin-mediated regulation of hepatic VLDL production
Source: JCI Insight. 2023 Aug 22;8(16):e166905. doi: 10.1172/jci.insight.166905 (PMC10543721; doi:10.1172/jci.insight.166905)
Supplement: Supplemental data [file jciinsight-8-166905-s213.pdf]

SUPPLEMENTAL MATERIAL

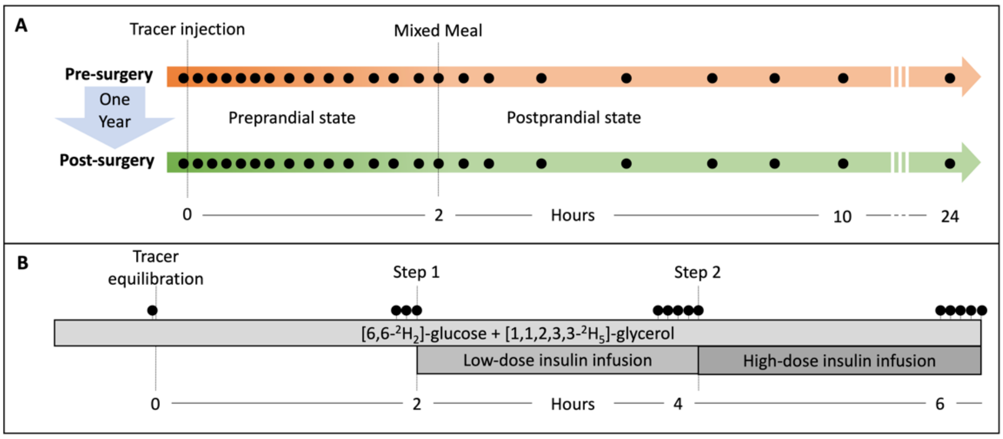

Figure S1: Schematic overviews of the lipoprotein kinetics experiment (A) and hyperinsulinemic euglycemic clamp (HEC) (B). For lipoprotein kinetics studies and HEC same procedures were followed before and one-year post-surgery. In both panels horizontal axes represent time-course of the experiment, where black dots indicate blood sampling.

Computational modeling

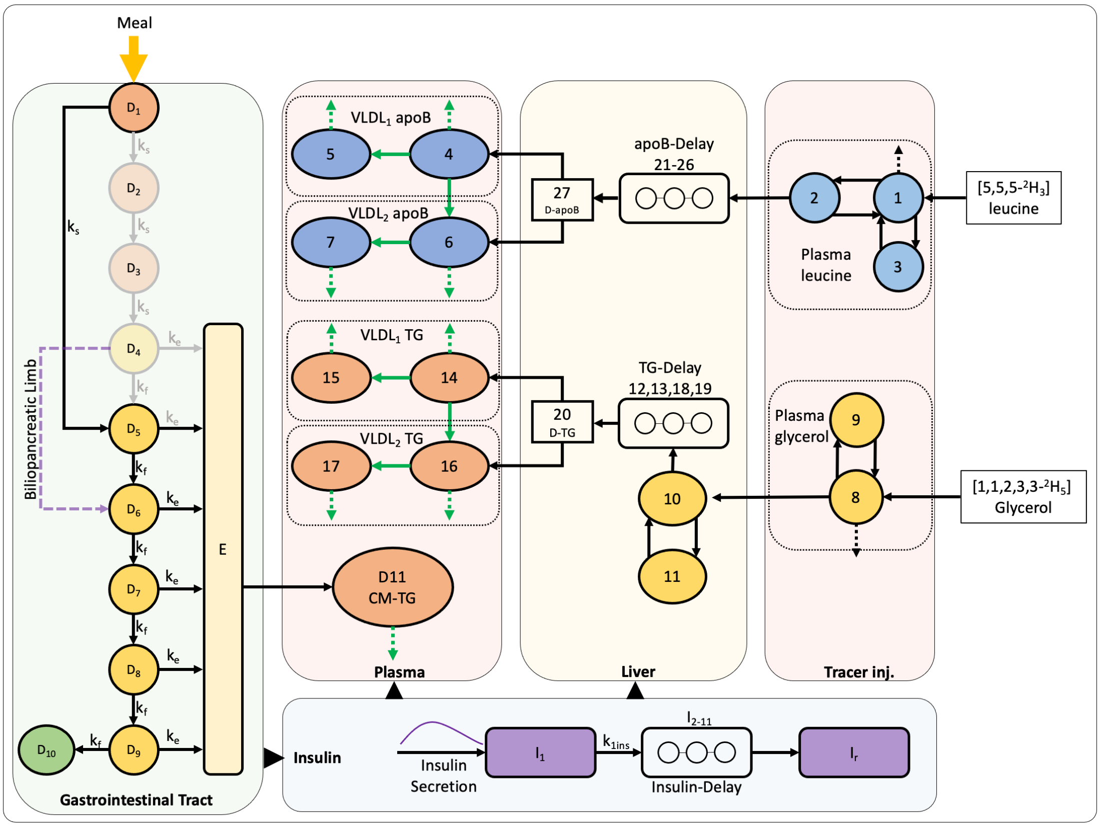

Figure S2: The key components and pathways of the computational model network. The model consists of gastrointestinal, plasma, liver, tracer injection and insulin modules. The letter D is used to indicate the compartments of the gastrointestinal tract module, whereas letter I is used to indicate compartments of the insulin module. The faded nodes within the gastrointestinal module indicate bypassed stomach and small intestinal compartments after bariatric surgery. Solid arrows represent transfer between corresponding compartments. Dotted arrows represent removal of material. Green arrows denote putative insulin-dependent stimulation. The red arrow represents putative insulin-mediated inhibition. The biliopancreatic limb, which is represented by the purple dashed arrow from

D_4 to D_6, is a passive pathway and depicted for illustration purposes. Abbreviations: $D_{1\text{-}3}$: stomach, $D_{4\text{-}9}$: small intestine, D_{10}: colon, E: common enterocyte pool, CM: chylomicron, I_1: plasma insulin, I_r: insulin response unit, TG: triglycerides (D-TG, D-apoB: distribution units).

In the multicompartmental model, the transfer rate from compartment *i* to *j* is given by non-negative coefficient *k(j,i)*. If there is no transfer from compartment *i* to compartment *j*, then *k(j,i)* is set to 0. Consequently, the rate of change of material in compartment Q_i is given by;

$$\frac{dQ_i}{dt} = \sum_{j\neq i} k(i,j)Q(j) - \sum_{i\neq j} k(j,i)Q(i) + U(i) \quad \text{(S1)}$$

where, the first sum represents the total flux into the compartment Q_i from other compartments and second sum represents outflux from the compartment Q_i. This system can be represented by the following system of differential equations;

$$\frac{dQ}{dt} = K \cdot Q + U \quad \text{(S2)}$$

where, *U* is the input, *Q* is the vector of model compartments or the state variables, and *K* is the matrix that holds transfer rate coefficients. The elements on the main diagonal of *K* are the negative sums of the elements in the corresponding column. Being defined this way, the diagonal element gives the negative of the total outflux rate from the corresponding compartment.

$$K = \begin{bmatrix} -\sum_{i\neq 1} k(i,1) & \cdots & k(1,n) \\ \vdots & \ddots & \vdots \\ k(n,1) & \cdots & -\sum_{i\neq n} k(i,n) \end{bmatrix} \quad \text{(S3)}$$

Modeling tracer injection, liver and plasma modules

Tracer injection, liver and plasma modules are represented by a 28 compartments system (Fig. S2), which is adapted from a previous model (Adiels et al., 2005) and adjusted to account for the postprandial regulations. Compartments 1 and 8 represents free plasma leucine and glycerol, respectively, where the labeled metabolites are injected. Plasma glycerol is converted to TG using the molar weight ratio of 885/92 g/g (Adiels et al., 2005) and the fractional leucine content of apoB is taken as 0.01212g/g (Demant et al., 1998). Leucine and glycerol are taken up by the liver and secreted as VLDL apoB and TG, respectively. The hepatic processing in the leucine and glycerol pathways are represented by 7 and 5 consecutive transient delay compartments, respectively (compartments 12,13,18,19,20 for glycerol and compartments 21-27 for leucine). The transfer coefficients between leucine and glycerol delay compartments are set to $7/\tau_l$ and $5/\tau_g$, respectively. After the delay, produced apoB is secreted into the plasma either as $VLDL_1$ or $VLDL_2$ (compartments 4 and 6, respectively). The fraction of apoB secreted into the compartment 4 is d_{4_27}, and thus the fraction that is secreted into the compartment 6 is $(1\text{-}d_{4\_27})$.

In the model, each plasma apoB compartment is coupled to a TG compartment. If the ith compartment represents an apoB pool, then the $i{+}10^{th}$ compartment is chosen to represent the associated plasma TG compartment for ease of illustration. The fractional transfer coefficients between plasma VLDL apoB compartments are defined in the classical way. This is, coefficient for apoB transfer from compartment *i* to *j* is defined as *k(j,i)*. The coefficient for the corresponding TG transfer from $i{+}10^{th}$ compartment to the compartment *j+10* is *k(j+10, i+10)*, which is proportional to *k(j,i)*. During this transfer, due to the TG hydrolysis, only a certain fraction of the TG that leaves compartment *i* reaches to the compartment *j*. If this fraction is given by $f_{j,i}$, then $k(j+10,i+10)=k(j,i).f_{j,i}$. The fraction of the TG that is removed from the circulation during this transfer is $(1- f_{j,i})$.

TG flows into the compartment 16 is either from compartment 20 or from compartment 14. The particles transferred from compartments 20 and 14 into the compartment 16 should have the same apoB/TG ratio. If the flux from compartment *i* to *j* is defined by *F(j,i)=k(j,i)Q(i),* this constraint can be imposed by setting *F(16,14)/F(6,4)=F(16,20)/F(6,27)* at the steady state. This leads to the following distribution term;

$$d_{14\_20} = \frac{d_{4\_27}}{d_{4\_27}+(1-d_{4\_27})f_{6,4}} \tag{S4}$$

Hence, the fraction of the TG flux from compartment 20 to the compartments 14 and 16 are given by d_{14_20} and $(1-d_{14\_20})$, respectively. The compartment 28 is the 'exit' compartment, which has no physiological meaning.

In order to calculate the enrichments, the same liver, tracer injection and plasma modules are used for tracer and tracee subsystems. This way, each tracee compartment coupled to a tracer counterpart. This raises the total number of compartments for liver, tracer injection and plasma modules to 56. The first 28 compartments represent the tracee subsystem as shown above (Q) and remaining 28 compartments are used to represent the tracer subsystem (q) in the following form,

$$\frac{dq}{dt} = K \cdot q + u \tag{S5}$$

where q is the vector of tracer compartments, K is the matrix that holds transfer rates that is defined above and u is the external input, which represents the labeled leucine and glycerol injections. This way, the enrichment of the metabolite in compartment i is calculated as the ratio of $q(i)$ and $Q(i)$.

Gastrointestinal module

Two hours after the tracer injection, patients were asked to consume a liquid mixed meal. Nutritional content of the mixed meal is given in Table S1. In the model, digested lipids are absorbed and incorporated into chylomicrons (CM) in the form of TG.

Table S1: Content of the mixed meal.

	**Quantity (gr)**	**Energy (kcal)**	**Tot. carb (gr)**	**Sugar (gr)**	**Protein (gr)**	**Tot. Fat (gr)**
**Fresubin Drink**	400	600	49.6	28	40	26.8
**Palmitate shake**	79.4	363.6	0.4	0.1	0.5	40.6
**Total**	479.4	963.6	50	28.1	40.5	67.4

The gastrointestinal module is represented by an 11 compartments system (Fig. S2), where the compartments are labelled with the latter *D*. Compartments $D_{1\text{-}3}$ represent stomach, $D_{4\text{-}9}$ represent small intestines. Lipid absorption and TG secretion into the plasma is only allowed from intestinal compartments. Colon is represented with a single closed compartment (D_{10}) and used to calculate the amount of unabsorbed lipids. Compartment D_{11} represents plasma chylomicron TG concentration, which links gastrointestinal module to the hepatic module through competition for clearance. Two hours after the tracer injection, patients were asked to consume a liquid mixed meal, which consisted of two servings of Fresubin® Protein Energy Drink enriched with a palmitate shake (Table S1). Due to the reduced stomach size, it took longer for patients to consume the meal after the surgery. Therefore, the meal input into the first stomach compartment D_1 is represented with a piecewise continuous square wave function in the following form;

$$\frac{dD_1}{dt} = m(t) - k_s D_1 \tag{S6}$$

$$m(t) = \begin{cases} 0, & 0 \leq t < 2 \\ \frac{m_{TG}}{t_c}, & 2 \leq t < t_c \\ 0, & t_c \leq t \end{cases} \tag{S7}$$

where m_{TG} is the approximate amount of TG that can be produced from consumed lipids, t_c is the average time it took for patient to consume the meal in hours and it is set to 0.25 and 0.5 hours for pre- and post-surgery conditions, respectively. For converting consumed lipids to TG and calculating m_{TG}, it was assumed that 100gr TG contained 95gr fatty acids, as suggested before (Ratnayake & Galli, 2009).

In the post-surgical configuration, the stomach is reduced to a single compartment (D_1) to account for the reduced stomach size, and this compartment is directly attached to the second intestinal compartment to represent the bypassed duodenum. In Figure S2, bypassed stomach and intestinal compartments after the surgery are shown with faded colors, where biliopancreatic limb is only shown for illustration purposes. The rate of change of the amount off lipids in gastric compartment D_i is given by the following differential equation,

$$\frac{dD_i}{dt} = k_s D_{i-1} - k_s D_i \tag{S8}$$

Half gastric emptying time ($T_{1/2}$) following a liquid test meal in healthy humans was measured as 80 minutes on average, with no correlation with age, sex or BMI (Hellmig et al., 2006; Mishima et al., 2009). Taking this as a reference, and solving system of equations given by S8 for all three gastric compartments simultaneously, parameter k_s is estimated as 2.005 h^{-1}. Furthermore, using identical transfer and absorption rate coefficients for intestinal compartments (k_f and k_e, respectively) and assuming a known malabsorption coefficient, the relation between intestinal lipid absorption and transfer rates, k_e and k_f, can be established in the following form;

$$k_e = k_f \left[\frac{1 - m_a^{\frac{1}{d_c}}}{m_a^{\frac{1}{d_c}}}\right], \tag{S9}$$

where, m_a is the malabsorption coefficient and d_c is the number of intestinal compartments for pre and post-surgery configurations. The malabsorption coefficient is calculated by the ratio of the lipids in the column to the total amount of consumed lipids. We used the values estimated by Odstrcil et al. for pre- and post-surgery lipid malabsorption coefficient as, 8% and 27% respectively (Odstrcil et al., 2010). Same absorption coefficient is used for all intestinal segments, except for the first intestinal compartment in the post-surgical configuration (D_5, the roux-limb). A separate parameter was employed for the lipid absorption from this compartment. However, the estimated value of this parameter approached to 0 during the model fit. Therefore, it was fixed to 0 in the final model.

Plasma lipoprotein remodeling and competition

In circulation, lipoproteins compete for the clearance pathways (Adiels et al., 2012; Brunzell, Hazzard, Porte, & Bierman, 1973; Cohn et al., 1993; Sondergaard et al., 2012; Van Beek et al., 1998). Studies show that larger lipoproteins have a higher affinity for LPL (Bjorkegren et al., 1996; Karpe & Hultin, 1995; Kovrov, Landfors, Saar-Kovrov, Näslund, & Olivecrona, 2022). Therefore, a larger lipoprotein has a higher chance for binding to the LPL and hence a higher clearance rate compared to the smaller particles (Adiels et al., 2012; Fisher, Coppack, Humphreys, Gibbons, & Frayn, 1995; Kovrov et al., 2022; Toyota, Yamamura, Miyake, & Yamamoto, 1999; Van Beek et al., 1998). In the pre-prandial state the system is at the steady state and the transfer rates are constant. However, in the postprandial state, the transfer rates dynamically respond to the altered pool sizes.

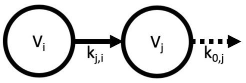

The diagram above shows a simple two compartment system to illustrate the transfer dynamics between model compartments. In the model, the rate of change of material in the compartment V_j is given by the following differential equation;

$$\frac{dv_j}{dt} = k_{j,i}v_i - k_{0,j}v_j \tag{S10}$$

Here, $k_{j,i}$ is the transfer rate of material from compartment V_i to compartment V_j and defined as follows;

$$k_{j,i} = \bar{k}_{j,i}p_j \tag{S11}$$

where, $\bar{k}_{j,i}$ is the constant transfer rate and p_j is the affinity of lipoprotein species V_j to the LPL. The binding probability of lipoprotein species V_j to LPL depends the ratio of its size to the average lipoprotein particle size in circulation. Since $VLDL_1$, $VLDL_2$ and chylomicrons are triglyceride rich lipoproteins, we assume that the size of each particle is proportional to its TG content. In the pre-prandial state, plasma concentrations are constant. Therefore, the affinity for each lipoprotein species (p_j) should be fixed to 1 and the clearance rate should be solely determined by $\bar{k}_{j,i}$, which is estimated from the data. In the postprandial state, the affinity becomes a dynamically changing function of the ratio of the size of the corresponding particle to the average lipoprotein particle size in circulation. Consequently, increased particle size increases the affinity for the corresponding lipoprotein species, where increased total plasma TG concentration reduces its affinity through competition. This way, the clearance rate for each lipoprotein species depends on the concentration of other lipoproteins as well. Under these assumptions, the affinity of the particle V_j to LPL is given by,

$$p_j = \left[\frac{\frac{VTG_j}{VApoB_j}\frac{VApoB_{j,b}}{VTG_{j,b}}}{\frac{\sum_i^N VTG_i}{\sum_i^N ApoB_i}\frac{\sum_i^N ApoB_{i,b}}{\sum_i^N VTG_{i,b}}}\right]^{n_a}, \tag{S12}$$

where, VTG_j and $VapoB_j$ are the amount of TG and apoB in the V_j fraction, respectively. The subscript b is used for baseline values for the corresponding metabolite. By normalizing variables over their baseline values, p_i becomes a dimensionless parameter that is 1 during the steady state. The total plasma apoB concentration virtually remains the same in the post-prandial state. Therefore, the baseline and post-prandial total plasma apoB terms cancel out in the denominator. Finally, since the relation between volume and surface area is nonlinear, the term is raised to n_a^{th} power, which is left as a free parameter to be estimated from the data.

Modeling insulin action

Insulin module consists of 12 transient delay compartments, which are labelled with the letter *I* in Figure S2. Compartment I_1 represents plasma insulin concentration. Since the relation between plasma insulin and the components of the lipoprotein metabolism is captured as a unidirectional link, the plasma insulin is used as a forcing function in the model. In order to obtain a smooth and continuous plasma insulin curve, insulin input is approximated with the following bell-shaped Gaussian function;

$$f(t) = \alpha \cdot e^{\frac{-(t-\beta)^2}{\delta^2}} \tag{S13}$$

where t is time, α determines the height of the peak, β is the peak location and parameter δ determines the width of the curve. When used as input, this curve is flexible enough to generate the measured plasma insulin concentration. Using Eq. S13 as input, the rate of change of plasma insulin concentration is given as;

$$\frac{dI_1}{dt} = \alpha \cdot e^{\frac{-(t-\beta)^2}{\delta^2}} + k_{1ins} I_b - k_{1ins} I_1 \qquad \text{(S14)}$$

Here I_b is the basal insulin concentration in *pmol/L* and $k_{1ins}I_b$ enforces insulin concertation to remain at the basal level when there is no input from *f*. Since lipoprotein metabolism does not feedback onto the insulin module, the parameters of the insulin input function *f(t)* (α, β, δ) are separately estimated for each patient and used in the model. Estimated values for α, β and δ for the population average are given in parameter table below (Table S2). Parameter k_{1ins} is fixed to the population average 2 h^{-1} to ensure better control over continuous insulin input curve. This way, input to the first delay compartment is fixed and the delay associated with insulin action is controlled by the free delay parameter that was estimated in the model. Resulted plasma insulin concentration for each patient is given in Figure S3. As it seen from the Figure S3, method produces a good fit for each patient. Although, the method leaves out some of the dynamic properties, such as the biphasic responses produced by some patients, it must be noted that these small deviations would not have a potent effect in the response compartment due to the long delay.

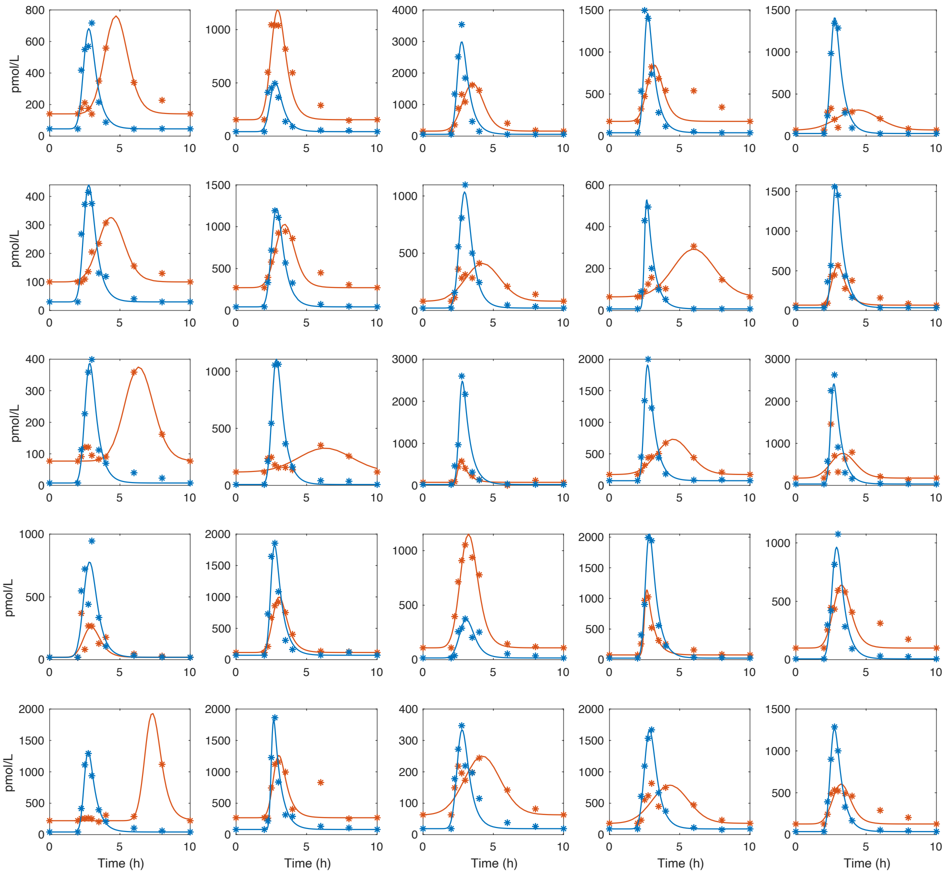

Figure S3: Pre- (red) and post-surgery (blue) plasma insulin concentrations simulated by the time dependent input function given in Eq. S13 (N=24). The last panel is the population average.

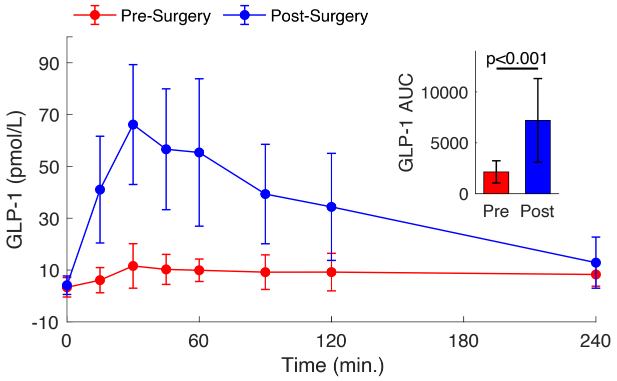

Figure S4: Pre (red) and post-surgery (blue) plasma active glucagon like peptide-1 (GLP-1) levels and area under the curve (AUC).

Insulin mediated LPL stimulation

Insulin regulates lipoprotein lipase at transcriptional and posttranslational levels (Fried, Russell, Grauso, & Brolin, 1993; Sadur & Eckel, 1982), which is compromised in insulin resistant patients (Panarotto, Rémillard, Bouffard, & Maheux, 2002). In the model, plasma insulin concentration is transmitted to the insulin response compartment (I_r) through a delay, which is given by 10 consecutive delay compartments ($I_{2\text{-}11}$). This delayed insulin response is then incorporated into the corresponding transfer coefficient given by Eq. S11, which reads as follows:

$$k_{j,i} = \left[1 + R_{max}\frac{(I_r - I_{rbase})}{(I_r - I_{rbase}) + K_{ins}}\right]\bar{k}_{j,i}p_j \qquad \text{(S15)}$$

where R_{max} is the maximal response that can be produced by insulin and it is fixed to 0.25 based on the approximate value obtained during a hyperinsulinemic clamp study (Sadur & Eckel, 1982). K_{ins} is the insulin responsiveness parameter and I_{rbase} is the response produced by the basal insulin concentration. Therefore, the term in the brackets in Eq. S15 is equal to 1 in the fasting state for all patients and it dynamically responds to the insulin change over basal level in the postprandial state. The potency of the insulin-mediated postprandial response depends on the insulin response parameter K_{ins}, which represents plasma insulin concentration over the basal level that is necessary to evoke the half maximal response, $R_{max}/2$. K_{ins} is left a free parameter and estimated for each patient from the data, where $1/K_{ins}$ was added to the cost function as a regularization term to prevent K_{ins} to converge arbitrarily small values. A smaller K_{ins} value corresponds to a higher insulin responsiveness. On the other hand, for very large K_{ins} values the term in the square brackets in Equation 1 approaches to 1, which indicates that there is no detectable dynamic insulin-mediated lipoprotein lipolysis stimulation in the model. Hence, the reciprocal of K_{ins} is a measure of insulin-mediated stimulation of lipoprotein lipolysis. We introduce lipoprotein lipolysis insulin sensitivity index (lipoprotein lipolysis ISI) in arbitrary units as follows;

$$Lipoprotein\ lipolysis\ ISI = \frac{K_s}{K_{ins}} \qquad \text{(S16)}$$

where, K_s is a scaling factor and set to 20 to contain the calculated indices between 0 and 1 for convenience.

Insulin mediated suppression of the hepatic apoB production has also been incorporated into the model as a delayed forcing signal generated by the portal vein insulin concentration, where portal vein insulin signal was derived from the plasma insulin data. However, insulin-mediated effects on the

hepatic apoB production pathway remained undetectable due to the uncertainty associated with portal vein insulin concentrations and resulting parameter estimates. Therefore, insulin mediated suppression of the hepatic apoB production was removed from the final version of the model.

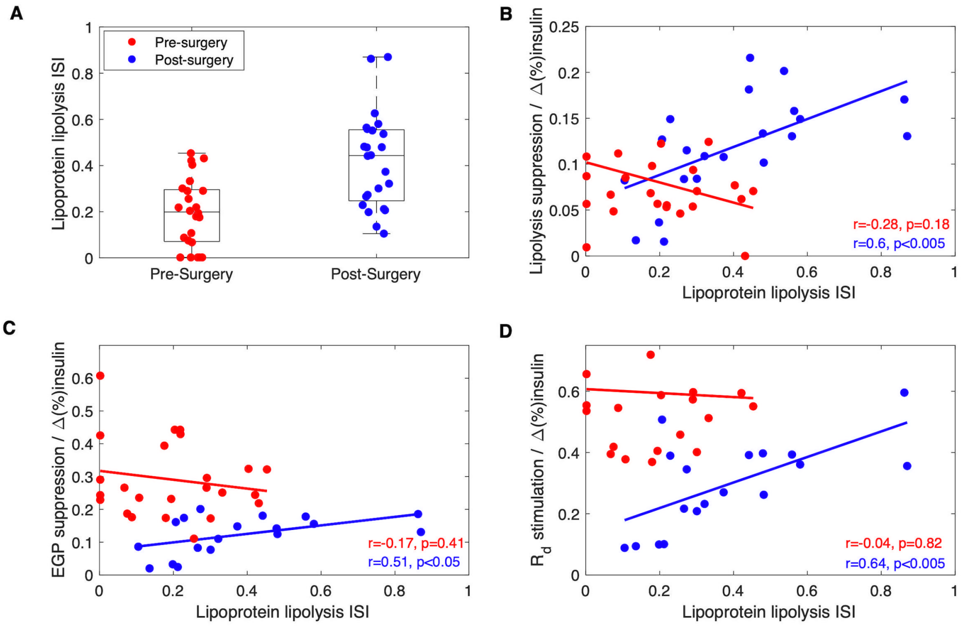

Figure S5: (A) Pre- and post-surgery lipoprotein lipolysis insulin sensitivity index (lipoprotein lipolysis ISI). (B) Pre- and post-surgery insulin mediated peripheral lipolysis suppression per insulin increased over basal level (Δ(%)insulin) from the clamp studies vs lipoprotein lipolysis ISI. (C) Pre- and post-surgery insulin mediated endogenous glucose production (EGP) suppression per Δ(%)insulin vs lipoprotein lipolysis ISI. (D) Pre- and post-surgery insulin mediated glucose rate of disappearance (R_d) stimulation per Δ(%)insulin vs lipolysis ISI. In each panel, the regression lines for the pre- and post-surgery data are shown in red and blue, respectively. Correlation coefficients (r) and associated p-values are reported in each panel.

Parameter Estimation and calculating production, fractional catabolic and transfer rates

Kinetic rate parameters were estimated by employing MATLAB's optimization toolbox. For each patient, model parameters were estimated by minimizing a cost function, defined as the weighted sum of squared differences between model simulation and the data. For the model simulations reported in Figures 1 and 2 reported in the results section in the main text, the computational model was calibrated using population average data and obtained parameter values are given in Table S2. During the parameter estimation process, multiple searches were initiated from randomly selected points in the parameter space. The parameter search resulting in the minimum error was selected as the optimal parameter set.

For model calibration and analysis following data is used; plasma $VLDL_{1,2}$ apoB and TG concentrations, plasma $VLDL_{1,2}$ apoB and TG enrichments, plasma chylomicron TG concentration, plasma leucine and glycerol enrichments, plasma insulin concentration. For each patient, parameter estimation is carried out by minimizing a cost function, which is defined as the sum of squared differences between model simulation and the data. The model consists of a system of ordinary differential equations that governs the rate of changes of internal state variables in the following form:

$$\dot{\vec{x}}(t) = f\left(\vec{x}(t), \vec{u}(t), \vec{\theta}\right) \quad \text{(S17)}$$

$$\vec{y}(t) = g\left(\vec{x}(t), \vec{\theta}\right) \quad \text{(S18)}$$

where $\vec{x}$ is the vector of the internal state variables, $\vec{u}$ is the vector of external inputs and $\vec{\theta}$ is the vector of model parameters (e.g. rate constants, transfer coefficients, initial conditions and scaling factors), and $\vec{y}(t)$ is the measurements. In the model, the first 28 entries of the vector $\vec{x}(t)$ represents the tracee subsystem (*Q*) given with the hepatic, plasma and tracer injection modules in Figure S1. *Q* represents unlabeled biochemical concentrations. State variables $x_{29\text{-}56}$ represent the tracer subsystem (*q*), where variable *q(i)=x(i+28)* corresponds to the tracer counterpart of the tracee compartment *Q(i)*. State variables $x_{57\text{-}68}$ represent gastrointestinal tract (*D*). Finally, variables $x_{69\text{-}80}$ represent insulin subsystem (*I*).

Since the system in hand is partially observed, measurements $\vec{y}(t)$ can be performed on a limited number of state variables and their certain combinations. Namely, plasma $VLDL_{1,2}$ apoB and TG concentrations, plasma $VLDL_{1,2}$ apoB and TG enrichments, plasma chylomicron TG concentration, plasma leucine and glycerol enrichments, plasma insulin concentration. In eq. S18, *g* is the mapping that associates internal state variables to the observables $\vec{y}(t)$ with scaling parameters in $\vec{\theta}$. The plasma biochemical concentrations are the sum of the corresponding compartments in the tracee subsystem. For instance, plasma $VLDL_2$ TG concentration is given by *Q(16)+Q(17)*. In the other hand, the enrichment of the corresponding metabolite is given by the tracer to tracee ratio (TTR), which is the ratio of the sum of the tracer and tracee compartments. For instance, the TTR for the plasma $VLDL_2$ TG is calculated by the following;

$$VLDL_{2,TG,TTR} = \frac{q(16)+q(17)}{Q(16)+Q(17)} \quad \text{(S19)}$$

The model parameters ($\vec{\theta}$) are estimated by minimizing the difference between model simulations and experimental data, which is measured by the cost function $J(\vec{\theta})$ that calculates the weighted sum of squared differences between model simulation and measured data;

$$J(\vec{\theta}) = \sum_{j=1}^{m} \sum_{k=1}^{T} \frac{\left(y_{D,j}(t_k) - y_j(\vec{\theta}, t_k)\right)^2}{\sigma_{D,j,k}} \quad \text{(S20)}$$

where $y_{D,j}(t_k)$ *is* the data for the jth observation measured at the kth time point and $\sigma_{D,j,k}$ is the measurement error associated with this data point. In the parameter estimation process, the parameter space Θ is searched for the parameter values that minimize the cost function, $J(\vec{\theta})$. For normally distributed error with zero mean this corresponds to the maximum likelihood estimate (MLE). During the parameter estimation process, multiple searches were initiated from randomly selected points in the parameter space generated by Latin Hypercube Sampling, and MATLAB's nonlinear optimization function *lsqnonlin* was used. The parameter search that was resulted in minimum error was selected as the optimal parameter set, $\vec{\theta}^*$. In order to evaluate the uncertainty associated with the estimated parameters, asymptotic confidence intervals were calculated by using the covariance matrix $C=F^{-1}$, where *F* is the Fisher Information matrix, and given by the negative hessian of the log-likelihood function calculated at the optimum point. This provides information about the curvature of $J(\vec{\theta})$ in the neighborhood of the optimum point $\vec{\theta}^*$, where a flat profile in the direction of a certain parameter indicates unidentifiability and results in an infinite confidence bound. We approximated the hessian of the log-likelihood by the first order model sensitivities given by the Jacobian matrix that is calculated during the parameter estimation, $H=J^TJ$ (Vanlier, Tiemann, Hilbers, & van Riel, 2013). The parameter confidence interval for the ith parameter is given by;

$$\theta_i^{\pm} = \theta_i \pm t_{\alpha,df}\sqrt{C(i,i)} \quad \text{(S21)}$$

where, $t_{\alpha,df}$ is the α quantile of the t-distribution with df degrees of freedom. Estimated model parameters and calculated confidence intervals for the population data are provided in Table S2.

Table S2: Pre and post-surgery estimated parameter values for population average data.

Parameter	Pre	Post
d_{4_27}	0,64 ± 0,02	0,49 ± 0,02
τ_l (h)	0,49 ± 0,02	0,52 ± 0,02
τ_g (h)	0,25 ± 0,03	0,26 ± 0,02
$f_{5,4}$	0,59 ± 0,13	0,16 ± 0,12
$f_{6,4}$	0,34 ± 0,02	0,35 ± 0,02
$f_{7,6}$	0,44 ± 0,1	0,07 ± 0,1
k(28,1) (h^{-1})	3,41 ± 0,09	2,79 ± 0,07
k(28,4) (h^{-1})	1,93 ± 0,82	1,95 ± 0,85
k(28,5) (h^{-1})	0,01 ± 0	0,01 ± 0
k(28,6) (h^{-1})	0,95 ± 0,13	0,75 ± 0,12
k(1,2) (h^{-1})	1,37 ± 0,18	1,63 ± 0,26
k(1,3) (h^{-1})	4,99 ± 0,31	5,78 ± 0,47
k(3,1) (h^{-1})	4,04 ± 0,37	3,19 ± 0,45
k(5,4) (h^{-1})	0,02 ± 0,01	0,03 ± 0,01
k(6,4) (h^{-1})	0,58 ± 0,4	0,1 ± 0,51
k(12,10) (h^{-1})	0,08 ± 0,01	0,06 ± 0,01
k(10,8) (h^{-1})	2,99 ± 0,68	2,27 ± 0,63
k(10,11) (h^{-1})	0,02 ± 0,01	0,03 ± 0,01
k(11,10) (h^{-1})	1,38 ± 0,21	1,27 ± 0,21
k(21,2) (h^{-1})	0,03 ± 0,01	0,02 ± 0
plasmaGlycerol (mg/L)	155,05 ± 13,41	195,76 ± 13,72
plasmaLeucine (mg/L)	601,86 ± 15,82	678,72 ± 14,91
k(28,8) (h^{-1})	20,47 ± 1,93	15,51 ± 1,38
k(9,8) (h^{-1})	12,96 ± 1,3	13,87 ± 1,28
k(8,9) (h^{-1})	7,27 ± 1,29	7,63 ± 1
k(7, 6) (h^{-1})	0,07 ± 0,02	0,11 ± 0,02
k(28, 7) (h^{-1})	0,03 ± 0,01	0,04 ± 0
n_a	2,68 ± 0,24	2,25 ± 0,36
k_f (h^{-1})	0,42 ± 0,04	1,07 ± 0,04
k_d(12,11) (h^{-1})	7,98 ± 0,48	19,81 ± 0,79
k_{1ins} (h^{-1})	1,54 ± 0,76	1,96 ± 0,72
K_{ins}	67,4 ± 47,36	36,1 ± 13,85
α	853±597	1246±645
β	2,84±1,24	2,5±0,06
δ	0,62±0,49	0,23±0,09

(Pre: pre surgery estimated value ± confidence bound, Post: post-surgery estimated value ± confidence bound)

After model parameters were estimated for each patient, we calculated the production/secretion (PS), fractional catabolic (FCR) and fractional transfer rate (FTR) parameters by utilizing calculated pool sizes and kinetic transfer rates. For calculating each kinetic parameter, the sum of fluxes from associated compartments was divided by the sum of corresponding pool sizes. Flux from compartment *i* to *j* is calculated as *F(j,i)=k(j,i)Q(i)*. Afterwards, PS, FCR and FTR parameters were calculated by employing fluxes and pool sizes by the following equations;

$$VLDL_{1,ApoB,FCR} = \frac{F(6,4)+F(28,4)+F(28,5)}{Q(4)+Q(5)} \quad \text{(S22)}$$

$$VLDL_{2,ApoB,FCR} = \frac{F(28,6)+F(28,7)}{Q(6)+Q(7)} \quad (S23)$$

$$VLDL_{ApoB,FTR} = \frac{F(6,4)}{Q(6)} \quad (S24)$$

$$VLDL_{ApoB,FDC} = \frac{F(28,4)+F(28,5)}{Q(4)+Q(5)} \quad (S25)$$

$$VLDL_{1,TG,FCR} = \frac{F(16,14)+F(28,14)+F(28,15)}{Q(14)+Q(15)} \quad (S26)$$

$$VLDL_{2,TG,FCR} = \frac{F(28,16)+F(28,17)}{Q(16)+Q(17)} \quad (S27)$$

$$VLDL_{TG,FTR} = \frac{F(16,14)}{Q(16)} \quad (S28)$$

$$VLDL_{TG,FDC} = \frac{F(28,14)+F(28,15)}{Q(14)+Q(15)} \quad (S29)$$

$$VLDL_{1,ApoB,PS} = F(4,27) \quad (S30)$$

$$VLDL_{2,ApoB,PS} = F(6,27) \quad (S31)$$

$$VLDL_{1,TG,PS} = F(14,20) \quad (S32)$$

$$VLDL_{2,TG,PS} = F(16,20) \quad (S33)$$

$$VLDL_{ApoB,FCR} = \frac{F(6,4)+F(28,4)+F(28,5)+F(28,6)+F(28,7)}{Q(4)+Q(5)+Q(6)+Q(7)} \quad (S34)$$

$$VLDL_{TG,FCR} = \frac{F(16,14)+F(28,14)+F(28,15)+F(28,16)+F(28,17)}{Q(14)+Q(15)+Q(16)+Q(17)} \quad (S35)$$

Pre-surgery (Table S3) and post-surgery (Table S4) kinetic rates are calculated for each patient. Since the postprandial pool sizes and transfer rates dynamically change over time, postprandial kinetics were calculated for every 30 minutes and their averages over their respective time-courses were represented as the final value. Physiological parameters for the population averages were calculated and reported as mean ± standard deviation (sd).

References

Adiels, M., Matikainen, N., Westerbacka, J., Söderlund, S., Larsson, T., Olofsson, S. O., … Taskinen, M. R. (2012). Postprandial accumulation of chylomicrons and chylomicron remnants is determined by the clearance capacity. *Atherosclerosis*, *222*(1), 222–228. https://doi.org/10.1016/j.atherosclerosis.2012.02.001

Adiels, M., Packard, C., Caslake, M. J., Stewart, P., Soro, A., Westerbacka, J., … Borén, J. (2005). A new combined multicompartmental model for apolipoprotein B-100 and triglyceride metabolism in VLDL subfractions. *Journal of Lipid Research*, *46*(1), 58–67. https://doi.org/10.1194/jlr.M400108-JLR200

Bjorkegren, J., Packard, C. J., Hamsten, A., Bedford, D., Caslake, M., Foster, L., … Karpe, F. (1996). Accumulation of large very low density lipoprotein in plasma during intravenous infusion of a chylomicron-like triglyceride emulsion reflects competition for a common lipolytic pathway. *Journal of Lipid Research*, *37*(1), 76–86. Retrieved from http://www.ncbi.nlm.nih.gov/pubmed/8820104

Brunzell, J. D., Hazzard, W. R., Porte, D., & Bierman, E. L. (1973). Evidence for a common, saturable, triglyceride removal mechanism for chylomicrons and very low density lipoproteins in man. *Journal of Clinical Investigation*, *52*(7), 1578–1585. https://doi.org/10.1172/JCI107334

Cohn, J. S., Johnson, E. J., Millar, J. S., Cohn, S. D., Milne, R. W., Marcel, Y. L., … Schaefer, E. J. (1993). Contribution of apoB-48 and apoB-100 triglyceride-rich lipoproteins (TRL) to postprandial

increases in the plasma concentration of TRL triglycerides and retinyl esters. *Journal of Lipid Research*.

Demant, T., Mathes, C., Gütlich, K., Bedynek, A., Steinhauer, H. B., Bosch, T., … Warwick, G. L. (1998). A simultaneous study of the metabolism of apolipoprotein B and albumin in nephrotic patients. *Kidney International*, *54*(6), 2064–2080. https://doi.org/10.1046/j.1523-1755.1998.00204.x

Fisher, R. M., Coppack, S. W., Humphreys, S. M., Gibbons, G. F., & Frayn, K. N. (1995). Human triacylglycerol-rich lipoprotein subfractions as substrates for lipoprotein lipase. *Clinica Chimica Acta*, *236*(1), 7–17. https://doi.org/10.1016/0009-8981(95)06032-3

Fried, S. K., Russell, C. D., Grauso, N. L., & Brolin, R. E. (1993). Lipoprotein lipase regulation by insulin and glucocorticoid in subcutaneous and omental adipose tissues of obese women and men. *Journal of Clinical Investigation*, *92*(5), 2191–2198. https://doi.org/10.1172/JCI116821

Hellmig, S., Von Schöning, F., Gadow, C., Katsoulis, S., Hedderich, J., Fölsch, U. R., & Stüber, E. (2006). Gastric emptying time of fluids and solids in healthy subjects determined by 13C breath tests: Influence of age, sex and body mass index. *Journal of Gastroenterology and Hepatology (Australia)*. https://doi.org/10.1111/j.1440-1746.2006.04449.x

Karpe, F., & Hultin, M. (1995). Endogenous triglyceride-rich lipoproteins accumulate in rat plasma when competing with a chylomicron-like triglyceride emulsion for a common lipolytic pathway. *J Lipid Res*.

Kovrov, O., Landfors, F., Saar-Kovrov, V., Näslund, U., & Olivecrona, G. (2022). Lipoprotein size is a main determinant for the rate of hydrolysis by exogenous LPL in human plasma. *Journal of Lipid Research*, *63*(1), 100144. https://doi.org/10.1016/j.jlr.2021.100144

Mishima, Y., Amano, Y., Takahashi, Y., Mishima, Y., Moriyama, N., Miyake, T., … Kinoshita, Y. (2009). Gastric emptying of liquid and solid meals at various temperatures. *Journal of Gastroenterology*, *44*(5), 412–418. https://doi.org/10.1007/s00535-009-0022-1

Odstrcil, E. A., Martinez, J. G., Santa Ana, C. A., Xue, B., Schneider, R. E., Steffer, K. J., … Fordtran, J. S. (2010). The contribution of malabsorption to the reduction in net energy absorption after long-limb Roux-en-Y gastric bypass. *American Journal of Clinical Nutrition*, *92*(4), 704–713. https://doi.org/10.3945/ajcn.2010.29870

Panarotto, D., Rémillard, P., Bouffard, L., & Maheux, P. (2002). Insulin resistance affects the regulation of lipoprotein lipase in the postprandial period and in an adipose tissue-specific manner. In *European Journal of Clinical Investigation* (Vol. 32, pp. 84–92). https://doi.org/10.1046/j.1365-2362.2002.00945.x

Ratnayake, W. M. N., & Galli, C. (2009). Fat and fatty acid terminology, methods of analysis and fat digestion and metabolism: A background review paper. *Annals of Nutrition and Metabolism*. https://doi.org/10.1159/000228994

Sadur, C. N., & Eckel, R. H. (1982). Insulin stimulation of adipose tissue lipoprotein lipase. Use of the euglycemic clamp technique. *Journal of Clinical Investigation*, *69*(5), 1119–1125. https://doi.org/10.1172/JCI110547

Sondergaard, E., Sorensen, L. P., Rahbek, I., Gormsen, L. C., Christiansen, J. S., & Nielsen, S. (2012). Postprandial VLDL-triacylglycerol secretion is not suppressed in obese type 2 diabetic men. *Diabetologia*, *55*(10), 2733–2740. https://doi.org/10.1007/s00125-012-2624-z

Toyota, Y., Yamamura, T., Miyake, Y., & Yamamoto, A. (1999). Low density lipoprotein (LDL) binding affinity for the LDL receptor in hyperlipoproteinemia. *Atherosclerosis*, *147*(1), 77–86. https://doi.org/10.1016/S0021-9150(99)00166-5

Van Beek, A. P., Van Barlingen, H. H. J. J., De Ruijter-Heijstek, F. C., Jansen, H., Erkelens, D. W., Dallinga-Thie, G. M., & De Bruin, T. W. A. (1998). Preferential clearance of apoB-48-containing lipoproteins after heparin- induced lipolysis is modulated by lipoprotein lipase activity. *Journal of Lipid Research*, *39*(2), 322–332. https://doi.org/10.1016/s0022-2275(20)33894-3

Vanlier, J., Tiemann, C. A., Hilbers, P. A. J., & van Riel, N. A. W. (2013). Parameter uncertainty in biochemical models described by ordinary differential equations. *Mathematical Biosciences*, *246*(2), 305–314. https://doi.org/10.1016/j.mbs.2013.03.006
